# Radiation-induced network rewiring in cancer: systems-level insights into environmental radioactivity and precision oncology

**DOI:** 10.3389/fbinf.2026.1876523

**Published:** 2026-07-20

**Authors:** Marckasagayam Priyadharshini, Saravanan Sekaran

**Affiliations:** Saveetha Institute of Basic Medical Sciences (SIBMS), Saveetha Institute of Medical and Technical Sciences, Saveetha University, Chennai, Tamil Nadu, India

**Keywords:** DNA damage response, good health and wellbeing, ionizing radiation, multi-omics integration, precision oncology, radiosensitivity, regulatory networks, systems biology

## Abstract

The impact of ionising radiation (IR) from environmental and therapeutic sources is complex and extends beyond the damage to DNA. These perturbations are conveyed by regulatory networks, such as transcription factors, microRNAs, long non-coding RNA, and signaling cascades, which in the end affect the susceptibility, progression, and treatment response to cancer. The traditional reductionist approaches, which are very useful, are not enough to explain emergent properties of these dynamic systems. The system’s biology paradigm has a great potential of providing the explanation of the rewiring of cellular networks by radiation and identify novel targets to be targeted in precision oncology. The review summarizes the current literature on radiation-induced alterations of network patterns and on the remodelling of the molecular landscape of cancer cells due to exposure to environmental radioactivity and therapeutic radiation exposure. We create awareness of methodological progress in the development and analysis of radiation-responsive regulatory networks, identify key hub molecules and pathways, and discuss the translation potential of using radiation-responsive regulatory networks in individual radiotherapy. This review is different from previous reviews that have concentrated on single mechanisms in single-omics viewpoints or focused on a single radiation type as environmental or therapeutic, since it brings together network topology analysis, multi-omics data and clinical translatability in the context of both types of radiation exposure. We explore how weaknesses in radiation-sensitive components of a tumor can be used to target radiation-resistant ones while at the same time, radiation-induced damage to normal tissues can be reduced by network-aware approaches.

## Graphical Abstract

**Figure:**
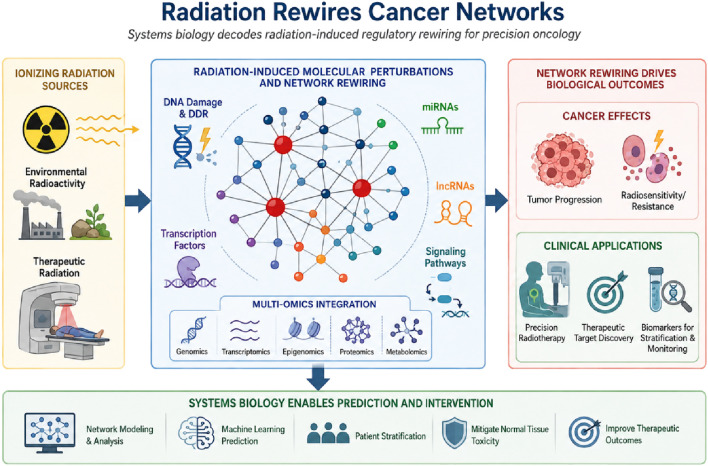

## 1 Introduction

From cosmic rays, terrestrial radionuclides, medical imaging and therapeutic uses, ionizing radiation is a part of our environment. The chronic low dose radiation exposure from both natural and anthropogenic source of radioactivity also affect the genomic stability and cellular signalling networks (Abbasi et al., 2026; Maghimaa et al., 2025; Ullah et al., 2026). Although low dose environmental exposures are associated with the risk of cancer in general, therapeutic radiation is a major pillar of oncology as ∼50% of cancer patients receive radiation therapy during their cancer treatment course (van Gisbergen et al., 2021). The biological effects of IR are mediated by a cascade of molecular events: direct ionization of DNA, generation of Reactive Oxygen Species (ROS) and activation of damage response pathways which govern cell fate: repair, senescence or death (Mzizi et al., 2024).

Radiation response is not a linear pathway, but rather an emergent property of interconnected networks within cells. Genes, proteins and non-coding RNAs can interact in complex feedback loops and perturbations at any node can cascade up predictably or unpredictably. This is one reason for the diversity of radiation sensitivity among different types of cancer and among people with cancer. The therapeutic ratio, the fundamental challenge of the therapeutic ratio, is the understanding of the manner in which these networks are rewired by radiation that is essential to develop strategies for increasing tumor radiosensitization while sparing normal tissues (van Gisbergen et al., 2021).

The conceptual and computational tools of systems biology can help to meet this challenge. Systems approaches can be used to model radiation-responsive networks, identify critical hubs and bottlenecks, and predict emergent behaviors not discernible by a single-gene based analysis, by combining transcriptomic, proteomic, metabolomic, and epigenomic data (Graw et al., 2021). The review focuses on methodological innovations, major molecules involved, and the translational relevance of radiation-induced alterations in the regulatory networks of cancer cells in precision radiotherapy. In the context of the increasing interest in radiation biology and systems level analysis, one of the major knowledge gaps is the lack of an integrated framework to connect molecular network rewiring, multi-omics data, and actionable clinical insights for precision radiotherapy. Previous reviews have generally focused on either molecular mechanisms or computational approaches. To fill this void, the current review combines network-based approaches and mechanistic insights of radiation responses to show the value of systems biology in directly informing radiation therapy. The integrated view is particularly timely in the light of recent developments in single cell profiling, space-omics, and multi-omics data integration. The key signaling pathways and network regulators central to this synthesis are summarized in Table 1.

**TABLE 1:** Key signaling pathways and network regulators in radiation-induced cancer rewiring

Pathway/Network	Key components	Functional role	Clinical relevance	References
DNA Damage Response	ATM, ATR, CHK1/2	DNA damage sensing and repair	Radiosensitization targets	Ciccia and Elledge (2010)
p53 signaling	p53, MDM2	Cell cycle arrest, apoptosis	Mutation → resistance	Vousden et al. (2009)
PI3K/AKT/mTOR	AKT, mTOR	Survival, anti-apoptosis	Therapy resistance	Porta et al. (2014)
MAPK pathway	ERK, JNK, p38	Stress response, proliferation	Tumor progression	Wagner et al. (2009)
NF-κB pathway	RelA, IκB	Inflammation, survival	Metastasis, resistance	Karin et al. (2009)

## 2 Radiation-induced DNA damage and repair are mediated by molecular mechanisms

### 2.1 Types of DNA lesions

It is responsible for various types of DNA damage including: base alteration, abasic, single strand breaks (SSBs), and especially double strand breaks (DSBs) (Ward, 1994). The complexity of the lesions depends on the linear energy transfer (LET) of the radiation source; most lesions created by low-LET radiation (X-rays, gamma rays) are simple, whereas high-LET particles (carbon ions, alpha particles) cause clustered damage, which is more difficult to repair (Mzizi et al., 2024). About 1,000 SSBs and 40 DSBs are generated per cell per gray of low-LET radiation (Murashko et al., 2021).

### 2.2 DNA damage response pathways

The sensor kinases that sense DSBs are the ATM (ataxia-telangiectasia mutated) and ATR (ATM and Rad3-related) kinases, which sense replication stress . Depending on the cellular context and extent of damage, these kinases trigger cell cycle arrest, DNA repair or apoptosis (Mzizi et al., 2024).

DSB repair can occur through two basic pathways: non-homologous end joining (NHEJ) is an error-prone pathway that operates throughout the cell cycle, and the other pathway is homologous recombination (HR) which relies on sister chromatid template and is active only during S and G2 phases (Lieber, 2008; San Filippo et al., 2008). These pathways are relatively abundant depending on the cell cycle phase and genetic context, resulting in repair fidelity and radiosensitivity. Mutations in either pathway, as is common in most cancers, opens opportunities of therapeutic exploitation.

### 2.3 The p53-MDM2 axis

P53 is the primary regulator of radiation response, regulating the expression of genes of cell cycle checkpoints, apoptosis and DNA repair. On IR exposure, p53 is stabilized and its transcription represses MDM2, a negative regulator of p53, which creates a negative feedback loop that regulates the duration and strength of the damage response (Haupt et al., 1997). MDM2 amplification and/or MDM2 overexpression alters this balance, resulting in radioresistance (Mzizi et al., 2024). The binding of p53 to MDM2 is a clinically proven therapeutic target and certain inhibitors of MDM2 including Nutlin-3 have radiosensitizing properties in preclinical models (Vassilev et al., 2004).

## 3 Radiation response non-coding RNAs

### 3.1 MicroRNAs as radiation-responsive regulators

MicroRNAs (miRNAs) are small non-coding RNAs (∼22 nucleotides) that are involved in the post-transcriptional regulation of mRNA stability and translation. Many miRNAs are expressed differently following IR exposure and changes in their expression regulate the activity of the DDR pathway, cell cycle progression and apoptosis (O'Brien et al., 2018). The regulatory networks as identified by Chauhan et al. (2022) that integrate miRNAs with radiation-responsive genes revealed that MDM2 and E2F7 are regulatory hubs and miR-25–3p, let-7a-5p and miR-497–5p have high betweenness centrality indicating that they can be critical regulators of information flow.

There are a number of miRNA families whose roles in radiosensitivity are well-characterized. The let-7 family, which is commonly suppressed in cancer, is a target of oncogenes such as RAS and MYC; its expression results in the enhancement of radiation-induced apoptosis (Weidhaas et al., 2007). On the other hand, radioresistance is promoted by miR-21 overexpression which inhibits PTEN and activates PI3K/AKT survival signaling (Gwak et al., 2012).

### 3.2 Non-coding RNAs in radionuclides response

The new DDR regulators are long non-coding RNAs (lncRNAs) that are transcripts over 200 nucleotides in length with no protein-coding capacity (Laurent et al., 2015). LncRNAs can also function as competing endogenous RNAs (ceRNAs) that bind to the miRNAs and release the bound mRNAs. A number of lncRNAs are also found to be altered in expression after radiation treatment and their activity has functional significance in the activity of the repair pathways and cell survival rates (Shaw and Gullerova, 2021).

For instance, LINC-ROR contains miR-145, which is known to stabilize the mRNA of the tumour suppressor p53, thus inhibiting the apoptosis of radiation-sensitive cells (Zhang and Peng, 2015). On the contrary, the lncRNA HOTAIR promotes the radioresistance of cervical cancer by epigenetic silencing of the gene p21 (Jing et al., 2015). The formation of cross-talk between these regulatory layers and the identification of nodes of potential therapeutic interest is the formation of integrated miRNA-lncRNA-mRNA networks, as seen in Chauhan et al. (2022).

The molecular mechanisms mentioned above, DNA damage and repair (Section 2) and non-coding RNA regulation (Section 3) are not independent of each other. Instead, they create connected nodes in regulating networks of bigger size whose emergent properties dictate cell fate after radiation exposure. Systems biology brings the tools of computation and conceptual analysis to the map and analyze these networks, leading from observation of individual molecules to network-level insights into action.

## 4 Systems biology approaches to radiation-responsive networks

### 4.1 Multi-omic data integration

This renders the radiation response a complex phenomenon that will need multi-level (integrative) mechanisms to achieve. By integrating Multi-omics, such as transcriptomics, proteomics, metabolomics and epigenomics, one can map the whole radiation-induced perturbations. Integration of multiple omics (transcriptomics, proteomics, metabolomics, epigenomics) allows for the complete mapping of radiation-induced perturbations (Graw et al., 2021). More powerful and less susceptible to false discovery Consensus methods that involve processing of multiple independent datasets to detect reproducibly altered molecules (Chauhan et al., 2022).

Chauhan et al. (2022) created a workflow to construct regulatory networks on radiation-exposed peripheral blood samples, using curated databases of TF-gene, miRNA-gene, and lncRNA-miRNA interactions. This method led to the identification of 20 DEGs that were reproducible across four independent data sets and these DEGs were extended into a network of 106 interactions among 11 DEGs, 78 miRNAs, 1 TF and 10 lncRNAs. These representations, based on a network, allow the detection of hub molecules and bottlenecks of pathways, which are not visible in single-gene analyses. For the sake of clarity, it is useful to give a brief overview of commonly used bioinformatics tools and data sources. Transcriptomic data of radiation-exposed cells and tissues are publicly available, and can be downloaded from databases like ArrayExpress and GEO (Gene Expression Omnibus). The data about interactions for network construction are taken from curated databases such as miRNA-gene (miRTarBase), TF-gene (ENCODE), and lncRNA-miRNA (LncBase). Network construction and visualization is usually done with Cytoscape and hub identification is done with the centrality metrics calculated in NetworkAnalyzer or igraph. Platforms like WGCNA, iCluster and MOFA (Multi-Omics Factor Analysis) are commonly used for multi-omic integration. The importance of hub validation across datasets has been reiterated and tools like GEO2R or DESEQ2 provide cross-dataset reproducibility analysis. The multi-omics categories, methods, and computational tools commonly used in this field are summarized in Table 2.

**TABLE 2:** Multi-omics and computational approaches for radiation-induced network analysis.

Category	Method/Tool	Application	Key insight	References
Genomics	WGS/WES	Mutation analysis	Radiation-associated signatures	Alexandrov et al. (2013)
Transcriptomics	RNA-seq	Gene expression profiling	Radiation-responsive genes	Rieger et al. (2004)
Proteomics	Mass spectrometry	Protein-level signaling	Pathway activation	Mertins et al. (2016)
Epigenomics	ChIP-seq, methylation	Chromatin regulation	Epigenetic rewiring	Ayrapetov et al. (2014)
Metabolomics	LC-MS	Metabolic profiling	Metabolic adaptation	Rabbani et al. (2010)
Network tools	Cytoscape, STRING	Network visualization	Interaction mapping	Shannon et al. (2003), Szklarczyk et al. (2021)
AI/Modeling	WGCNA, Bayesian, Deep learning	Network inference	Hub identification, prediction	Langfelder et al. (2008), Needham et al. (2007), LeCun et al. (2015)

### 4.2 Network topology and hub identification

There are several measures available for network analysis to determine biologically important nodes. The hubs with many partners have degree centrality, which is the number of connections to a node. The nodes that are part of betweenness centrality have a high number of the shortest paths connecting two others and can potentially be an information flow bottleneck (Abbasi et al., 2012). High betweenness centrality nodes may play out of proportion relative to their number of direct links in the network.

E2F7 seemed to be a hub node in radiation sensitive networks with high degree and centrality of betweenness (Chauhan et al., 2022). E2F7 is a transcriptional repressor that suppresses the expression of genes needed to enter S-phase and repairing, which may be considered a brake on proliferation following DNA damage (de Bruin et al., 2003). Its network location could imply that it can act as a point of integration between radiation-induced signals coordinating the transition between cell cycle arrest and proliferation resumption. In terms of the strength of such network results, the multiple complementary approaches of cross-dataset reproducibility (identifying hub genes in different independent transcriptomic datasets), functional validation (using siRNA knockdown or CRISPR perturbations to assess the phenotypic impact), and proteomic validation (Western blot or proteomic analysis to ensure transcriptomic results are recapitulated at the protein level) are commonly used. Chauhan et al. (2022) used cross-dataset reproducibility as a key filter and picked only the genes that were reproducibly altered in four independent datasets. This boosts confidence, but experimental validation of some of the key hubs, like E2F7 and MDM2, in specific radiation environments is a worthwhile next step for the field.

### 4.3 Computational modeling of radiation response

Besides the aforementioned networks mapping at rest, dynamical models can be employed to forecast changes in radiations responses over time. Kinsella (1996) proposed an approach to the radiosensitization of human tumors based on exploiting cell-cycle and DNA-repair differences between tumor and normal tissue, providing early conceptual groundwork for treating radiation response as a manipulable network property. Kinsella et al. (2011) used experiments and hybrid stochastic biochemical models to simulate the repair of the radiation damage by DNA mismatch repair. These can include cell cycle control, rates of DNA repair, and random changes in the amount of molecules available to make cell fates.

The bystander effect, dynamics of tumor oxygenation and clonogenic survival have been simulated with agent-based models and ordinary differential equation systems (ODES) (Powathil et al., 2016). Radiosensitivity prediction using machine learning methods which are trained using multi-omic data can predict radiosensitivity based on the molecular profile (Yard et al., 2016).

## 5 Metabolic reprogramming and radiation response

### 5.1 The warburg effect and radiation resistance

Aerobic glycolysis (the Warburg effect) is one of the defining features of cancer cells which prefer this metabolic pathway even in the presence of oxygen in the cell (Warburg, 1956). This metabolic phenotype provides biosynthetic precursors for proliferation and also it provides reducing equivalents (NADPH) to neutralize oxidative stress, such as ROS produced by radiation (van Gisbergen et al., 2021).

The hypoxia-inducible factor (HIF-1) is an overall controller of metabolic response, as it upregulates glycolytic enzymes and downregulates mitochondrial respiration in hypoxic environments. Hypoxia in tumors is a well-established determinant of radioresistance as molecular oxygen is needed to fix radiation-induced ROS on DNA damage (Gray et al., 1953). Even in normoxic conditions, stabilization of HIF-1alpha can thus also play a role in treatment resistance.

### 5.2 Targeting metabolism for radiosensitization

Radiosensitization can be done in the event of metabolic vulnerabilities. Complex I inhibitors (Metformin, Phenformin, Papaverine): These inhibitors decrease oxygen consumption and result in increase in the effect of radiation as hypoxia of the tumor is reduced (Zannella et al., 2013). Atovaquone is a complex III inhibitor that, in addition to its FDA approval for malaria prevention, is a comprehensive review of metabolic modulators with a radiosensitizing potential (Skwarski et al., 2021).

The other target is the metabolism of glutamine. It is common in many tumors to have what is known as glutamine addiction where the tumors become dependent on glutaminolysis to replenish the intermediates of the TCA cycle and maintain redox balance (Wise and Thompson, 2010). Telaglenastat (CB-839), a glutaminase inhibitor, increases radiation efficacy in preclinical models and is in clinical development (Gross et al., 2014).

## 6 Systems-level biomarkers for radiation exposure and response

### 6.1 Gene expression signatures

Typical transcriptional changes occur in the cells with radiation exposure, and may serve as markers for biodosimetry. In most studies and across various cell types, CDKN1A (p21), GADD45A and MDM2 are generally upregulated, consistent with a critical function in cell cycle arrest and in orchestration of DDR (Amundson et al., 2003). Chauhan et al. (2022) selected reproducibly radiation-responsive genes in peripheral blood as FDXR, DDB2, POLH and PCNA based on their roles in the biogenesis of iron-sulfur complexes, nucleotide excision repair, translesion synthesis and DNA replication, respectively.

Signatures of gene expression have been created to be used to estimate the dose in case of a radiation accident. Paul and Amundson (2008) showed that radiation doses as low as 0.5 Gy could be differentiated by transcriptional profiles against unexposed controls with potential use in the triage following radiological emergencies.

### 6.2 Protein biomarkers

The most popular molecular marker of DSBs is the phosphorylated form of histone variant H2AX, γH2AX, which is rapidly phosphorylated on serine 139 at sites flanking DNA double-strand breaks (Rogakou et al., 1998). Immunofluorescence microscopy is a sensitive assay of the DSB burden and repair kinetics of megabases chromatin domains flanking the break that can be used to detect the foci of γH2AX.

Inflammatory markers (IL-6, CRP), acute phase proteins (SAA, APOE) and stress-responsive enzymes (α-amylase) are other protein biomarkers (Mzizi et al., 2024). Serum amyloid A (SAA) and C-reactive protein (CRP) are markers of radiation-induced inflammatory responses which might cause late tissue toxicity. Multi-biomarker arrays, combinations of gene expression and protein markers, may be more powerful in estimating dose than individual markers.

### 6.3 Network-based biomarkers

The single-molecule biomarkers only represent a small part of the biological response to radiation. Methods based on networks may be more predictive (Chuang et al., 2007), and they can be based on a number of species of molecules, or a number of perturbations to networks. For example, rather than directly measure the expression of the individual DNA repair genes, it is possible to directly measure the expression of weighted combinations of the expression levels of individual genes in the pathway to measure the overall activity state of the HR or NHEJ pathway. Representative network-based biomarkers and their translational implications are summarized in Table 3.

**TABLE 3:** Network-based biomarkers and translational implications in radiation oncology.

Biomarker type	Example	Network role	Clinical application	References
Gene signatures	DNA repair genes (BRCA1, RAD51)	DNA repair efficiency	Predict radiosensitivity	Speers et al. (2015)
Hub proteins	AKT, p53	Central network regulators	Targeted therapy	Porta et al. (2014), Vousden and Prives (2009)
Pathway activity	PI3K/AKT signaling	Survival network activation	Patient stratification	Porta et al. (2014)
Immune markers	IL-6, TGF-β	Microenvironment signaling	Predict immunotherapy response	Schaue et al. (2010)

The precise genomic and molecular signatures detailed in Section 6 give the molecular underpinning for a precision oncology strategy for radiotherapy. The next section discusses the implications of these findings for the prediction of individual tumor response to radiation, selective targeting of network hubs for therapy, and the protection of normal tissues—all toward an optimized therapeutic ratio.

## 7 Implications for precision radiotherapy

### 7.1 Predicting radiosensitivity from molecular profiles

The response to radiation therapy varies between patients due to genetic, epigenetic and microenvironmental factors. Mutations in DNA repair genes (BRCA1/2, ATM), MMR deficiency and activation of oncogene (KRAS, EGFR) modulate radiosensitivity (Yard et al., 2016). Kinsella et al. (2011) showed increased MMR-deficient tumors incorporate more radiosensitizing thymidine analogs (IUdR) which suggests potential for using MMR status to select patients for radiosensitization with halogenated pyrimidines.

The multi-omic profiling integrated by machine learning algorithms has a higher prediction potential of radiosensitivity than individual biomarkers. The Cancer Genome Atlas (TCGA) produced huge molecular data sets of thousands of cancers, on which predictive models could be built (Yard et al., 2016). These models will have to be prospectively validated before they can be adopted into the treatment plan and put into clinical practice. In real world applications, network-based conclusions are starting to help stratify patients for radiotherapy. For instance, tumors with molecularly identified ATM mutations have changes in network connectivity in DNA damage response pathways and preclinical data suggests that these patients may be candidates for dose-escalation or combinations of ATM inhibitors. EGFR-amplified tumors, which feature hyperactivated survival signaling hubs, have also been targeted in clinical trials in combination with radiotherapy, as has been recently done in head and neck cancers, for instance, with the use of cetuximab and radiation. Finally, scores derived from a network of genes involved in the DDR have been suggested as means of dose adaptation, or giving a higher dose to predicted radioresistant tumors, but sparing patients predicted to be radiosensitive from toxicity. These examples demonstrate the process of taking systems-level molecular analysis from exploration to clinical decision making.

### 7.2 Targeting network hubs for radiosensitization

Network analysis determines nodes that perturbation can cause disproportionate changes to the behavior of the system. The high-degree hubs and high-betweenness bottlenecks can be considered the possible targets of radiosensitization strategies. The disruption of the interaction between MDM2 and p53 can inhibit MDM2 (Vassilev et al., 2004) by using small molecules such as Nutlin-3, idasanutlin.

Another approach to therapeutic intervention is through miRNA therapeutics. The use of antagomirs to inhibit oncogenic miRNAs (miR-21) and miRNA mimics to restore them (miR-21) is possible (Cortez et al., 2014). The choice of therapeutic development should be guided by their network attributes, which include the number of targets they are connected to, the number of target miRNAs that they have in their portfolio, as well as their betweenness and degree.

### 7.3 Protecting normal tissues

Not only is it possible to sensitize the tumors but also to protect the normal tissues, which enhances the therapeutic ratio. An example of radioprotector, Amifostine selectively collects in the normal tissues and eliminates free radicals produced by radiation (Kouvaris et al., 2007). Metabolic interventions can also be used as a means of selective protection of normal tissue: diets or a ketogenic diet can promote the differentiation of radiation resistance in normal tissues while keeping the radiation resistance of the tumor intact (Safdie et al., 2009; van Gisbergen et al., 2021).

Knowledge of the network topology of normal tissue radiation response could be used to identify other protective cell targets. GADD45A, a stress-inducible gene which has a role in cell cycle arrest and DNA repair is activated in both tumor and normal cells; strategies to selectively enhance its activity in normal tissues and impair it in tumors could improve the therapeutic ratio (Liebermann and Hoffman, 2008).

### 7.4 Challenges and limitations today

Although systems biology research has been advocated for radiation research, some significant issues need to be recognized. First, the complexity of data integration is a major challenge: the integration of transcriptomic, proteomic, metabolomic and epigenomic data is a difficult process, which involves careful normalization, batch correction and harmonizing among different data types with varying noise structures. Second, the issue of reproducibility is still a problem because hub genes and network topologies found in one dataset do not always replicate in another independent set of data, in part because of different cell types, x-rays, and time point of measurement. Third, most network models constructed are static snapshots, while radiation response is extremely dynamic, and multi-omic profiling in time is resource heavy and not routinely done in clinics. Fourth, there is a lack of prospective clinical trials that use network-based biomarkers, regulatory challenges to multiple gene biomarker assays, and a need for standardized bioinformatics pipelines. To meet these challenges, multi-institutional studies, open sharing of data and investments in computational infrastructure that can manage large-scale multi-omic studies are needed.

## 8 Future directions

### 8.1 Spatial and single-cell resolution

The bulk transcriptomic studies calculate the means of signals in heterogeneous cell populations masking significant subpopulation dynamics. Single-cell RNA sequencing (scRNA-seq) can be used to characterize cell-type-specific responses to radiation, and to identify resistant subclones (Tang et al., 2009). Spatial transcriptomics adds positional information, reflecting the impact of radiation on various components of the tumor microenvironment, such as well oxygenated perivascular areas, and hypoxic cores (Ståhl et al., 2016). Conventional bulk analyses suffer from certain restrictions that these technologies can overcome. scRNA-seq can identify rare radioresistant sub-populations that would be lost and undetectable in bulk data, and build cell-type-resolved radiation-response networks. For example, cancer stem-like cells, detected by scRNA-seq, might have unique hub gene expression in the DNA repair pathway which could serve as specific targets for radiosensitizing. Spatial transcriptomics can also be used to map the heterogeneity of response to radiation in the tumour microenvironment, where cells that are close to each other, such as those near the vasculature or immune infiltrates, are found to have different activation of networks. The combination of scRNA-seq and spatial data with network analysis tools is an intriguing field in radiation biology and will eventually aid spatially targeted radiotherapy dose painting: increasing the radiation doses to specific tumor areas with spatial radioresistance.

### 8.2 Temporal dynamics

Radiation response takes place over the timescale between seconds (first damage recognition) and days (cell fate commitment) up to years (carcinogenesis, late normal tissue effects). Most studies only document one time point and are missing key dynamic transitions. Dynamical modelling of multi-omic time-series data can explain the dynamics of networks and uncover important transition points that can be addressed by therapeutic interventions (Bar-Joseph et al., 2012).

### 8.3 Integration with immunotherapy

Radiation-induced immunogenic cell death (ICD) releases damage-associated molecular patterns as well as tumor antigens that can prime anti-tumor immunity (Apetoh et al., 2007). A new type of clinical interest is the use of immunotherapeutic and radiotherapy in combination with immune checkpoint inhibitors. To optimize combination approaches (Formenti and Demaria, 2012), radiation-immune interactions at systems-level will be necessary.

## 9 Conclusion

The effects of ionizing radiation are propagated through a chain of molecular events that further interact through interconnected regulatory networks that result in cell fate and tissue response. The tools that are necessary in understanding this complexity are systems biology approaches which combine multi-omic information, build and analyze network models, and simulate dynamical behavior. The network analysis identified key hub molecules and bottleneck nodes that can be considered as potential therapeutic targets of radiosensitization, such as MDM2, E2F7, miR-25–3p, let-7a-5p.

The methodology should be further developed, clinically validated in a prospective fashion and combined with new technologies like single-cell and spatial profiling to translate understanding into precision radiotherapy. Rational strategies can be developed to exploit vulnerabilities of the network in cancer while maintaining the interconnected networks of normal tissues. The key next steps are: to harmonise multi-omics workflows across radiation research; to set up prospective clinical trials based on biomarkers; and to create computational platforms for real-time integration of patient omics data into radiotherapy planning. The field also needs to consider the reproducibility and validation of hub genes identified in a variety of data sets and types of tumors. Optimization of the therapeutic ratio in radiation oncology will rely on systems biology as the analytical framework that emerges as these challenges are met.
